# Natural history and clinical assessment of Taiwanese patients with mucopolysaccharidosis IVA

**DOI:** 10.1186/1750-1172-9-21

**Published:** 2014-02-10

**Authors:** Hsiang-Yu Lin, Chih-Kuang Chuang, Ming-Ren Chen, Pao Chin Chiu, Yu-Yuan Ke, Dau-Ming Niu, Fuu-Jen Tsai, Wuh-Liang Hwu, Ju-Li Lin, Shuan-Pei Lin

**Affiliations:** 1 Department of Medicine, Mackay Medical College, New Taipei City, Taiwan; 2 Department of Pediatrics, Mackay Memorial Hospital, Taipei, Taiwan; 3 Department of Medical Research, Mackay Memorial Hospital, Taipei, Taiwan; 4 Mackay Junior College of Medicine, Nursing, and Management, Taipei, Taiwan; 5 Institute of Clinical Medicine, National Yang-Ming University, Taipei, Taiwan; 6 Medical College, Fu-Jen Catholic University, Taipei, Taiwan; 7 Institute of Biotechnology, National Taipei University of Technology, Taipei, Taiwan; 8 Department of Pediatrics, Kaohsiung Veterans General Hospital, Kaohsiung, Taiwan; 9 Department of Pediatrics, Taichung Veterans General Hospital, Taichung, Taiwan; 10 Department of Pediatrics, Taipei Veterans General Hospital, Taipei, Taiwan; 11 Department of Pediatrics, China Medical University Hospital, Taichung, Taiwan; 12 Department of Pediatrics, National Taiwan University Hospital, Taipei, Taiwan; 13 Division of Medical Genetics, Department of Pediatrics, Chang Gung Children’s Hospital, Taoyuan, Taiwan; 14 Department of Infant and Child Care, National Taipei University of Nursing and Health Sciences, Taipei, Taiwan

**Keywords:** Clinical manifestations, Diagnosis, History, Management, Mucopolysaccharidosis IVA

## Abstract

**Background:**

Mucopolysaccharidosis IVA (MPS IVA) is a rare lysosomal storage disorder caused by *N*-acetylgalactosamine-6-sulfatase deficiency, which catalyzes a step in the catabolism of glycosaminoglycans, keratan sulfate and chondroitin-6-sulfate. This disease has a variable age of onset and rate of progression.

**Methods:**

A retrospective analysis of medical records of 24 patients with MPS IVA (11 males, 13 females; current mean age ± SD, 12.6 ± 6.6 years; age range, 1.4-29.4 years) seen at 6 medical centers in Taiwan from January 1996 through June 2013 was performed.

**Results:**

Mean ages of onset of symptoms and confirmed diagnosis were 2.0 ± 1.6 and 5.7 ± 4.5 years, respectively. The most prevalent clinical manifestations were kyphosis (100%), pectus carinatum (96%), abnormal gait (93%), striking short trunk dwarfism (92%), genu valgum (92%), and valvular heart disease (91%). Eight patients (33%) experienced at least one surgical procedure with the most common being ear tube insertion (25%), adenoidectomy (17%), tonsillectomy (13%), supraglottoplasty (13%), spinal decompression (13%), and spinal fusion (13%). The most prevalent cardiac valve abnormalities were aortic stenosis (45%) and mitral regurgitation (45%). At the time of the study, 8 out of 24 patients (33%) have died at the mean age of 17.2 ± 7.7 years.

**Conclusions:**

An understanding of the natural history involved in MPS IVA may allow early diagnosis of the disease. All affected Taiwanese patients experienced significant functional limitations. Adequate evaluations and timely management may improve clinical outcomes and quality of life.

## Introduction

Mucopolysaccharidosis IVA (MPS IVA; OMIM 253000, Morquio A disease) is an autosomal recessive disorder caused by a deficiency of the lysosomal enzyme *N*-acetylgalactosamine-6-sulfatase (GALNS, EC 3.1.6.4), which catalyzes a step in the catabolism of glycosaminoglycans (GAGs), keratan sulfate (KS) and chondroitin-6-sulfate (C6S). It leads to accumulation of the KS and C6S, mainly in bone and cornea, causing a systemic skeletal dysplasia [1,2]. MPS IVA has a variable age of onset and rate of progression. Common presenting features include elevation of urinary and blood KS, short stature, odontoid hypoplasia, pectus carinatum, kyphosis, scoliosis, genu valgum, coxa valga, flaring of the lower ribs, hypermobile joints, corneal clouding, valvular heart disease, hearing loss, hepatomegaly, pulmonary compromise, coarse face, widely spaced teeth, and frequent caries; however, there is no central nervous system impairment. Odontoid hypoplasia is the most critical skeletal feature in MPS IVA patients, which in combination with ligamentous laxity and extradural GAG deposition, results in atlantoaxial subluxation, cervical myelopathy, paralysis, or even death.

Generally, MPS IVA patients with a severe form do not survive beyond the third decade of life primarily due to cervical instability or pulmonary compromise, whereas those patients with an attenuated form may survive over 70 years [3,4]. There has been no specific treatment for MPS IVA, and care has been palliative. Enzyme replacement therapy (ERT) has emerged as a potential treatment for MPS IVA [5].

The incidence of MPS IVA differs among different populations; reported rates range from 1 in 76,000 live births in Northern Ireland to 1 in 640,000 live births in western Australia [6,7]. In Taiwan, the incidence of MPS IVA is approximately 1 in 300,000 live births [8]. The concept of the founder effect may account for the discrepancy of the incidence in different ethnic populations.

There are only a handful of reports describing the natural history of MPS IVA [4,9,10]. The purpose of this study is to retrospectively collect and analyze the clinical and laboratory information recorded on the medical charts of Taiwanese MPS IVA patients, including medical history, clinical manifestations and assessments, diagnosis, and symptomatic management.

## Patients and methods

A retrospective study was performed for patients diagnosed with MPS IVA seen from January 1996 to June 2013 in six medical centers in Taiwan, including Mackay Memorial Hospital, Kaohsiung Veterans General Hospital, Taichung Veterans General Hospital, China Medical University Hospital, National Taiwan University Hospital, and Chang Gung Children’s Hospital. The diagnosis of MPS IVA was confirmed by leukocyte GALNS activity assay and/or two-dimensional electrophoresis of urinary GAGs [11]. Information on 24 patients (11 males and 13 females; mean age, 12.6 ± 6.6 years; age range, 1.4-29.4 years) was collected. Patients’ charts were reviewed for medical history, clinical manifestations and assessments, and laboratory studies. Findings on physical examinations were obtained from physicians’ records on the outpatient clinic or during admission. Each patient’s demographic information, including gender, age at onset of symptoms, age at confirmed diagnosis, age at and cause of death (if patient died), laboratory results, height, weight, body mass index (BMI), ambulatory status at the time of the latest medical records, physical examinations, and surgical interventions (if any) were recorded as applicable. Any available results of the following examinations were also collected, including 6-minute walk test (6-MWT), 3-minute stair climb test (3-MSCT), ophthalmologic evaluation for visual acuity and slit lamp examinations, electrocardiography (ECG), echocardiography, pulmonary function tests by spirometry, hearing assessment by pure-tone audiometry, bone mineral density (BMD) by dual energy x-ray absorptiometry (DXA), polysomnography, and other investigations as well as information relevant to the course of disease. Kaplan-Meier survival analyses were performed to calculate probability of survival. Standard deviation scores (*z* scores) for height, weight, and BMI were calculated using standard growth tables for Taiwanese population [12]. A *z* score was derived by subtracting the population mean from each individual’s raw score and then dividing the difference by the standard deviation (SD) of the population. Results are expressed as the mean ± SD unless otherwise indicated. ECG and echocardiographic examinations were carried out as previously described [13], and measurements were compared with normal data [14]. For pulmonary function assessment, forced vital capacity (FVC) and forced expiratory volume in 1 second (FEV_1_) were evaluated by standard spirometry techniques according to the American Thoracic Society guidelines [15-17]. For hearing assessment by pure-tone audiometry, the degree of hearing loss was classified by the age independent World Health Organization (WHO) clinical guidelines [18]. DXA was performed to assess BMD of the lumbar spine (L1-L4), using the Hologic QDR 4500 system (Bedford, MA, USA) [19]. Polysomnography was performed according to the guidelines of the American Thoracic Society. The obstructive apnea-hypopnea index (OAHI) was based on the number of obstructive/mixed apneas and hypopneas that occurred per hour of total sleep time [15,20]. Written informed consent was obtained from a parent for children and from patients over 18 years. The study was approved by the ethics committee of Mackay Memorial Hospital, Taipei, Taiwan.

### Statistical analysis

Descriptive statistics, including means and standard deviations, were calculated. The relationship between age and height, weight, and BMI of the 24 patients with MPS IVA was determined using Pearson’s correlation coefficient (*r*), and significance was tested using Fisher’s *r-z* transformations. All statistical analyses were performed using SPSS version 11.5 (SPSS Inc., Chicago, IL, USA), and differences with *p* < 0.05 were considered statistically significant.

## Results

**Figure 1:**
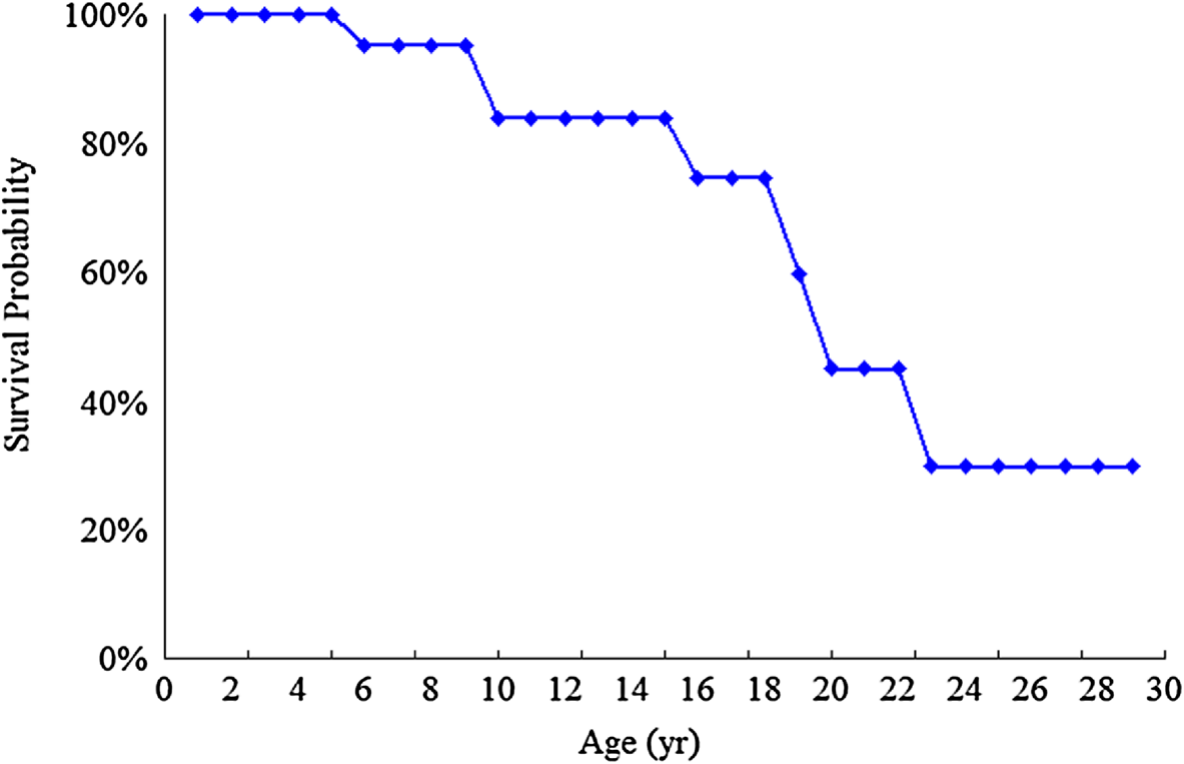
Kaplan-Meier Survival Curve for 24 Patients with MPS IVA in Taiwan.

**Figure 2:**
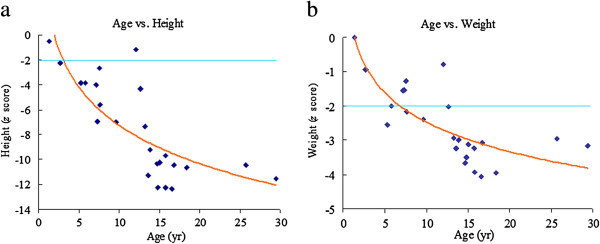
**Age against standard deviation scores (*z *scores) for height (a) and weight (b) of 24 patients with MPS IVA.** The values of the 2 parameters both decreased with age (*p* < 0.05). Horizontal lines represent the lower limits of normal.

**Figure 3:**
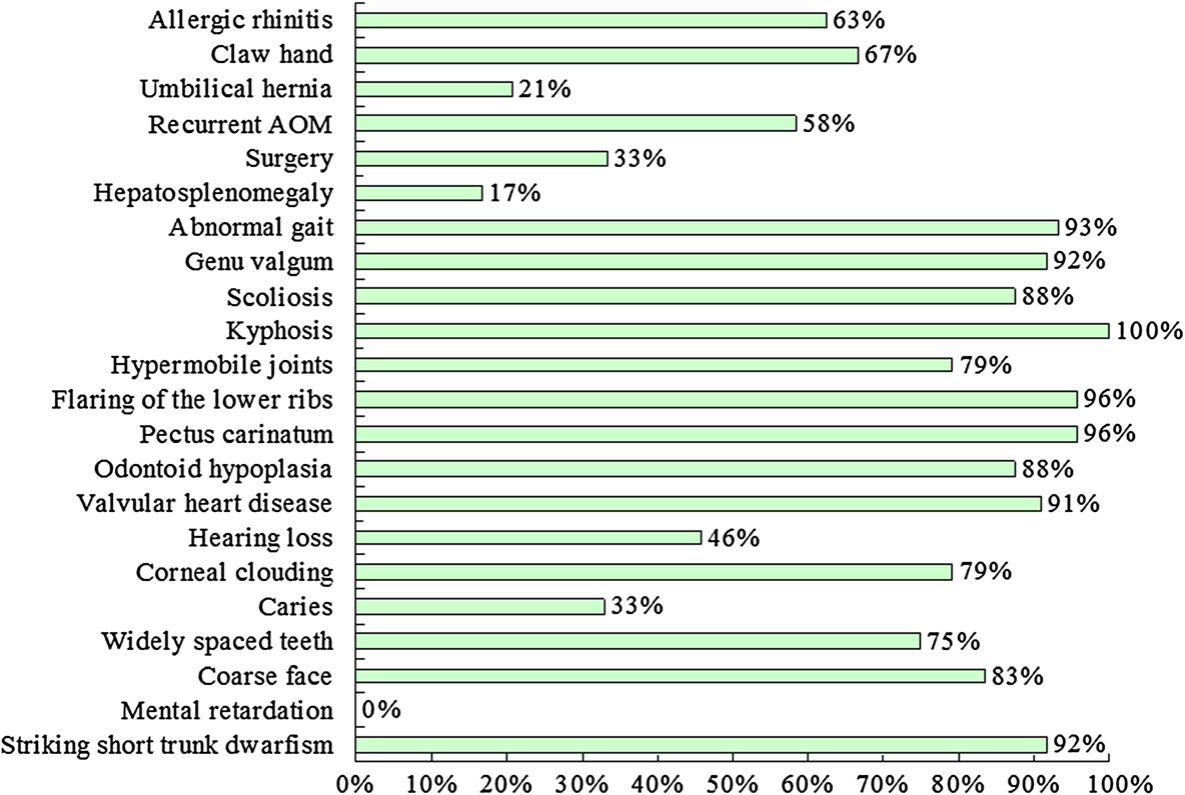
Clinical features of 24 patients with MPS IVA in Taiwan.

**Figure 4:**
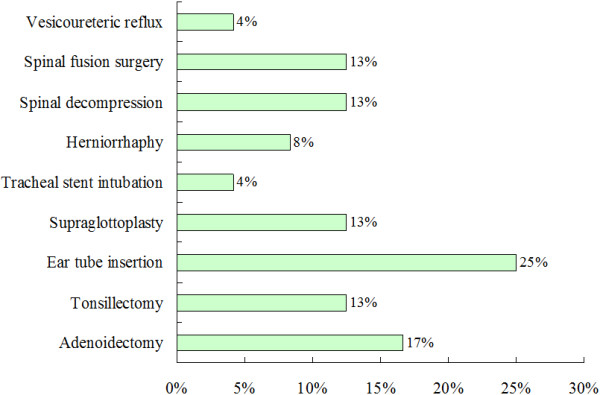
Types of surgical interventions for 24 patients with MPS IVA in Taiwan.

Table 1 showed the medical history, laboratory data, and clinical manifestations of 24 patients with MPS IVA in Taiwan. The mean ± SD ages of onset of symptoms and confirmed diagnosis were 2.0 ± 1.6 and 5.7 ± 4.5 years, respectively. The most common initial symptom was kyphosis or gibbus (92%, 22/24). The ambulatory status of these 24 patients was 13 (54%) with walking, 7 (29%) with wheelchair bound, and 4 (17%) with bedridden. For 11 patients who were able to perform the physical endurance tests, the mean ± SD values of 6-MWT and 3-MSCT were 235.3 ± 125.5 m and 31.0 ± 13.0 stairs/min, respectively. At the time of the present study, eight of 24 patients had passed away at the mean ± SD age of 17.2 ± 7.7 years (range 6.8-30.3 years). The cause of death was identified in 4 patients, while 4 patients died without a definite cause recorded. Figure 1 showed the Kaplan-Meier Survival Curve for these 24 patients. The survival probabilities at 5, 10, 15, 20, and 25 years were 100%, 84%, 84%, 45%, and 30%, respectively. Twenty-two patients (92%) had short stature and 17 (71%) were underweight with a *z* score of < -2. The mean *z* scores for height, weight, and BMI at the time of the latest medical records were -7.51 ± 3.86, -2.54 ± 1.09, and -0.1 ± 0.91, respectively. Both *z* scores for height and weight were negatively correlated with age (*r* = -0.769 and -0.693, respectively; *p* <0.01) (Figure 2). The most prevalent clinical manifestations were kyphosis (100%), pectus carinatum (96%), flaring of the lower libs (96%), abnormal gait (93%), striking short trunk dwarfism (height *z* score < -2) (92%), genu valgum (92%), and valvular heart disease (91%) (Figure 3). Eight patients (33%) experienced at least one surgical procedure. The most prevalent surgical interventions were ear tube insertion (25%), adenoidectomy (17%), tonsillectomy (13%), supraglottoplasty (13%), spinal decompression (13%), and spinal fusion (13%) (Figure 4).

**Table 1:** Medical history, laboratory data and clinical manifestations of 24 patients with MPS IVA in Taiwan

**Patient**	**Gender**	**Age (yr)**	**Age at onset of symptoms (yr)**	**Age at diagnosis (yr)**	**Initial symptoms**	**Leukocye GALNS activity (μmol/g protein/hr)a**	**Urinary GAG (μg/mg Creatinine)b**	**Height (z score)**	**Weight (z score)**	**BMI (z score)**	**Ambulatory status**	**6-MWT (m)**	**3-MSCT (stairs/min)**	**Visual acuity right/left eye**	**Age of passed away (yr)**	**Cause of death**
1	M	1.4	0.5	1	gibbus, kyphosis	0.03	519.1	-0.54	0.00	0.46	Walking	NA	NA	NA		
2	M	2.7	0.5	1.2	kyphosis	0.1	220.4	-2.25	-0.93	-0.42	Walking	NA	NA	NA		
3	M	5.3	0.4	1	gibbus	0.04	NA	-3.84	-2.54	-1.34	Wheelchair bound	NA	NA	NA	6.8	Atlantoaxial subluxation with spinal cord compression
4	F	5.8	0.3	3.5	pigeon chest, gibbus	0.77	NA	-3.84	-2.00	1.66	Walking	183.9	37	0.6/0.6		
5	F	7.2	2.4	2.5	chest cage deformity	0.51	445.6	-4.00	-1.56	0.51	Walking	219.3	15	0.2/0.3		
6	M	7.3	1	2.5	pigeon chest	0.3	218.5	-6.96	-1.53	2.64	Walking	225.9	28	0.4/0.4		
7	F	7.6	1.7	6.5	pigeon chest	0.09	191.5	-2.68	-1.28	-0.14	Walking	360	55	0.4/0.4		
8	F	7.7	2	8	kyphosis, chest cage deformity	0.4	228.5	-5.63	-2.18	0.29	Bedridden	NA	NA	NA	10.1	Unknown
9	M	9.6	0.5	5	spinal deformity, pigeon chest	0.5	NA	-6.98	-2.39	-0.47	Wheelchair bound	NA	NA	NA	10.8	Unknown
10	M	12.1	2.1	9	kyphosis, pigeon chest	0.043	251.1	-1.18	-0.78	-0.42	Wheelchair bound	NA	NA	0.6/0.6	19.4	Airway impairment
11	F	12.6	4	4	kyphosis	0.3	203.6	-4.35	-2.02	-0.85	Walking	125.7	15	1.0/1.0		
12	M	13.2	1.5	10	kyphosis, chest cage deformity	0.6	332.5	-7.35	-2.94	-1.35	Bedridden	NA	NA	NA	16.8	Unknown
13	F	13.6	3	3.1	kyphosis	0.3	333.6	-11.28	-3.25	0.05	Walking	495.9	48	0.3/0.4		
14	M	13.9	4.5	5	kyphosis, chest cage deformity	0.3	251.1	-9.20	-2.99	-0.53	Walking	147	24	NA		
15	F	14.7	4	4.3	kyphosis, chest cage deformity	0.07	323.0	-10.36	-3.67	-0.89	Bedridden	NA	NA	0.3/0.4		
16	F	14.8	0.4	0.4	kyphosis, chest cage deformity	0.1	179.4	-12.25	-3.49	0.11	Walking	140.7	37	0.3/0.9		
17	F	15.1	0.5	15	pigeon chest, prominent joints	0.7	451.3	-10.23	-3.13	-0.15	Bedridden	NA	NA	NA		
18	M	15.8	0.6	5	kyphosis	0.5	NA	-9.66	-3.24	-1.25	Wheelchair bound	NA	NA	0.5/0.6		
19	F	15.8	5.2	6	kyphosis, chest cage deformity	0.4	333.6	-12.27	-3.93	0.03	Walking	360	19	0.5/0.4		
20	F	16.6	3	6.6	short stature, knocked knees	0.172	222.8	-12.36	-4.06	-0.74	Wheelchair bound	NA	NA	NA	20.0	Airway impairment
21	F	16.8	1	1.5	kyphosis	0.3	NA	-10.45	-3.07	0.48	Walking	72.9	27	0.6/1.0		
22	F	18.4	4	14	kyphosis, chest cage deformity	0.5	NA	-10.66	-3.94	-0.66	Wheelchair bound	NA	NA	NA	23.1	Unknown
23	M	25.7	0.5	4.5	kyphosis	0.4	118.6	-10.46	-2.96	0.16	Walking	256.5	37	0.5/0.1		
24	M	29.4	4	17	joint contracture except small joints	0.11	103.6	-11.54	-3.16	0.31	Wheelchair bound	NA	NA	NA	30.3	Airway impairment
	Mean	12.6	2.0	5.7		0.3	273.8	-7.51	-2.54	-0.10		235.3	31.0		17.2	
	SD	6.6	1.6	4.5		0.2	113.6	3.86	1.09	0.91		125.5	13.0		7.7	

^a^Normal value: 5.9-27.8 nmol/h/mg protein.

^b^Normal values are age dependent: 1-3 years, 20.0-110.5; 3-5 years, 10.7-112.0; >5 years, 10.8-77.5.

GALNS, N-acetylgalactosamine-6-sulfate sulfatase; GAG, glycosaminoglycan; z score, standard deviation score; BMI, body mass index; 6-MWT, 6-minute walk test; 3-MSCT 3-minute stair climb test.

### Slit lamp examination by ophthalmologists

Visual acuity assessment by Snellen Fraction (n = 13) revealed that 9 of the 13 patients (69%) had normal right eye vision (20/63 or better), and 11 patients (85%) had normal left eye vision (20/63 or better). Ocular abnormalities in the 15 patients assessed included 14 patients (93%) with corneal clouding, 9 (60%) with astigmatism, 5 (33%) with hyperopia, 2 (13%) with myopia, and 1 (7%) with glaucoma.

### ECG and echocardiography

**Figure 5:**
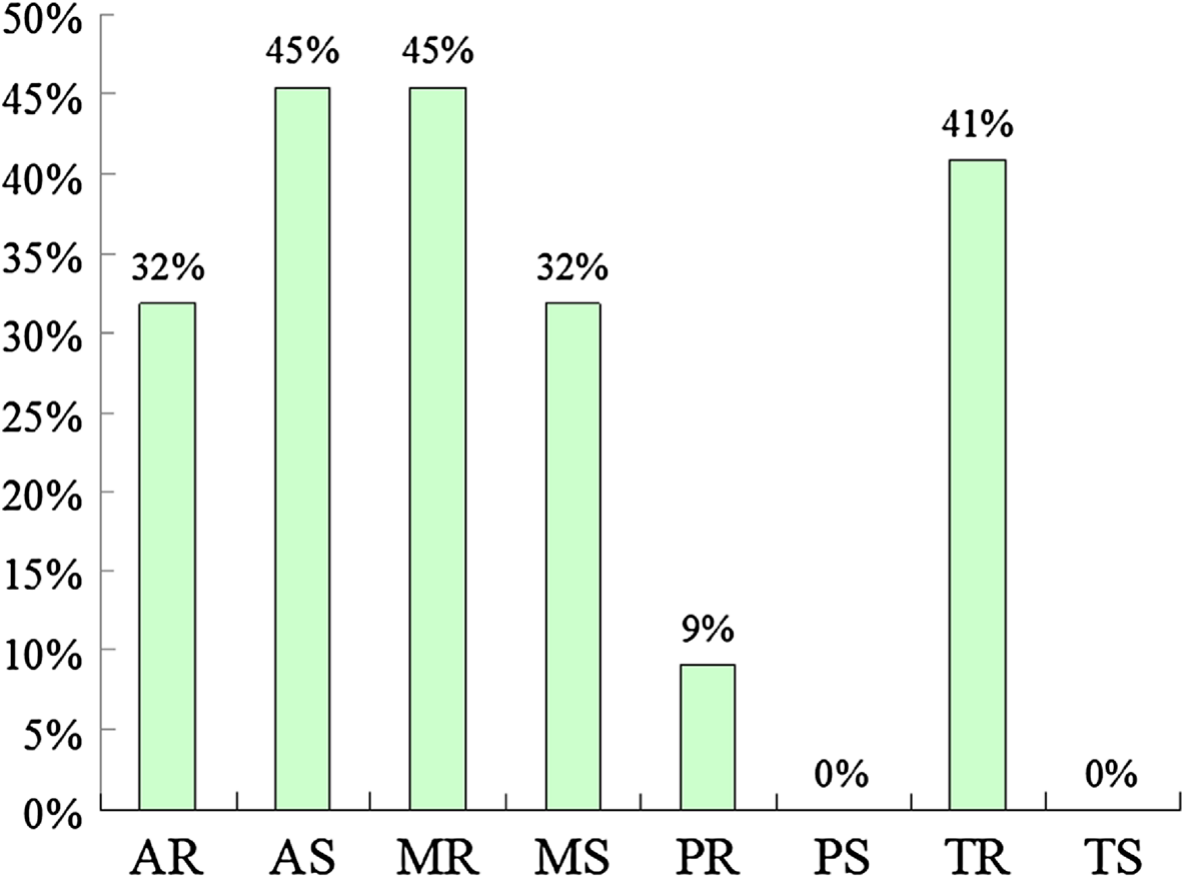
**Incidence of cardiac valve abnormalities in 22 patients with MPS IVA in Taiwan.** AR, aortic regurgitation; AS, aortic stenosis; MR, mitral regurgitation; MS, mitral stenosis; PR, pulmonary regurgitation; PS, pulmonary stenosis; TR, tricuspid regurgitation; TS, tricuspid stenosis.

The most common results from ECGs (n = 17) showed the presence of sinus arrhythmia (47%), sinus tachycardia (35%), and right or left axis deviation (18%). Echocardiographic examinations (n = 22) revealed that 20 patients (91%) had valvular heart disease. Sixteen (73%) and eleven patients (50%) had valvular regurgitation or stenosis, respectively. The most prevalent cardiac valve abnormalities were aortic stenosis (45%) and mitral regurgitation (45%), followed by tricuspid regurgitation (41%), aortic regurgitation (32%), mitral stenosis (32%), and pulmonary regurgitation (9%) (Figure 5). Nine patients (41%) had a thickened interventricular septum. The existence of the thickened interventricular septum was positively correlated with the increasing age (*r* = 0.690, *p* <0.01).

### Pulmonary function tests by spirometry

Thirteen patients had spirometric assessment performed to evaluate pulmonary function. By spirometric classification, 11 patients (85%) had small airway disease [mean forced expiratory flow during the middle half of FVC (FEF_25-75%_) < 65%], and 4 (31%) had restrictive lung disease (FEV_1_/FVC >0.7 with FVC <80% predicted).

### Hearing assessment by pure-tone audiometry

Hearing assessment by pure-tone audiometry was carried out in 9 patients. Mean values of air conduction (AC) and bone conduction (BC) of the better ear were 47.8 dB and 29.3 dB, respectively (normal hearing AC ≤ 25 dB and BC ≤ 20 dB). Mean air-bone gap was 20.3 dB (normal hearing air-bone gap < 15 dB). According to the WHO classification, 6 patients (67%) had various degrees of hearing loss (AC > 25 dB) with moderate (AC 41–60 dB) in 3, severe (AC 61–80 dB) in 2, and profound (AC ≥ 81 dB) in 1 patient. Three patients were classified with mixed type hearing loss, 1 with pure conductive type, 1 with pure sensorineural type, and 1 undefined.

### BMD assessment by DXA

Eight patients had DXA to access BMD. After correction for height-for-age *z* score (HAZ) as previously described [19], HAZ adjusted BMD *z* score was -0.72 ± 1.27. Three patients (38%) had osteopenia (HAZ adjusted BMD *z* score < -1 and ≥ -2) and 1 patient (13%) had osteoporosis (HAZ adjusted BMD *z* score < -2).

### Polysomnography

Overnight polysomnographic findings were available in 3 patients. They all had some degree of obstructive sleep apnea with mild (OAHI 1.5-5) in 1, and moderate (OAHI 5–10) in 2.

## Discussion

This is the first report to describe the natural history and clinical features of patients with MPS IVA in a single population. As far as we are aware, these were the only patients in Taiwan with confirmed diagnosis with MPS IVA at the time of this study. Accurate knowledge of the natural course of the disease will assist in evaluation of clinical and biomarker endpoints for clinical trials and to judge disease progression and therapeutic effects. The natural course and clinical manifestations of this disorder have been reported by the International Morquio A Registry and the Morquio A Clinical Assessment Program (MorCAP) [4,10]. Although the number of enrolled patients in these studies was as large as 326 and 325 subjects from 42 and 10 countries, respectively, the reports were collected from different ethnic populations, as well as analyzed altogether. Our results showed that Taiwanese patients with MPS IVA manifest a broad spectrum of disease phenotypes from mild to severe, indicating the clinical heterogeneity of the disease.

Information characterizing cause of death in MPS IVA is limited in the literature. In the report of Hunter Outcome Survey (HOS) [21], 46% (59/129) mortality was caused by airway impairment. Shih et al. [22] described that there were significant airway changes in the shape of the vocal cords and trachea in patients with MPS, which may be due to abnormal submucosal storage of GAG. Hendriksz et al. [23] reported that high mortality and morbidity rates in patients with severe MPS IVA are related to the occurrence of cervical myelopathy and dysplasia. Thus it was reasonable that 3 out of our 4 patients who died with a definitive cause were due to airway impairment, and the other was owing to atlantoaxial subluxation with spinal cord compression.

Montano et al. [4] reported that the mean ages of initial symptoms and confirmed diagnosis were 2.1 and 4.7 years, respectively, for patients in the International Morquio A Registry data. We had similar result with the age of initial symptoms being 2.0 years. However, the age of confirmed diagnosis in our series was delayed for 1 year (5.7 years).

In Montano’s data, 52% patients underwent surgical intervention, with the most frequent interventions being cervical spinal fusion and decompression (51%) and ear tube insertion (33%). In our data, only 33% experienced at least one surgical intervention with 25% being ear tube insertion, and only 13% undergoing cervical spinal fusion and decompression. This may be due to the mean age of patients in Montano’s data being 16.9 years, whereas mean age in our data was 12.6 years. Due to the progressive worsening of this disorder, the younger age of our patients may explain the lower percentage receiving operations. An alternative thought is that regular magnetic resonance imaging and prophylactic intervention are not part of clinical management in Taiwan compared with US, UK and part of Europe. Besides, the disease awareness of both medical providers and caregivers for the need for surgical procedures may also play a pivotal role for the discrepancy between these 2 data sets.

Short stature is one of the most characteristic features of MPS IVA. Ninety-two percent of our patients had striking short stature and 71% weight below normal for age with a *z* score of < -2. The mean *z* scores for height and weight were -7.51 ± 3.86 and -2.54 ± 1.09, respectively. The older patients had even lower *z* scores for height and weight, illustrating the progressive nature of the disease.

Montano et al. [4] described that 2/3 of their MPS IVA patients had hypermobility of joints, a unique feature to Morquio A syndrome caused by metaphyseal deformities and degradation of connective tissue near the joint, similar to the 79% reported in our study.

In our study population, 33% had dental caries and 75% had widely spaced teeth, consistent with James et al. [24] who reported that patients with MPS IVA have increased caries rates and enamel defects comparing with both patients with other MPS types and the general population.

Physical endurance measures are important indicators for evaluating functional impairment in subjects with progressive diseases [25]. The results of 6-MWT and 3-MSCT in the MPS IVA population show impairment regardless of age group [10]. In our study, only 11 patients (46%) were able to perform the physical endurance tests. The mean 6-MWT distance of 235.3 ± 125.5 m is significantly reduced as compared with the normal range of 470–664 m for unaffected children aged 4–16 years, and 500–580 m for healthy adults [26-28]. The mean 3-MSCT value of 31.0 ± 13.0 stairs/min also revealed a significant impairment comparing with the baseline stair climb rate of 50 ± 29.5 stairs/min in the Phase 2 study for the MPS VI patients. However, in the Phase 3 study, rates were significantly lower 30 and 19 on the placebo and active treatment groups [29].

Danes et al. [30] described that diffuse corneal clouding is the most common ocular finding in MPS IVA, although it occurs to a lesser extent and is more slowly progressive than in other MPS types. Couprie et al. [31] reported that patients with MPS IV tend to have astigmatism besides myopia and hyperopia. In our study subjects, 93% had corneal clouding, 60% had astigmatism, 33% had hyperopia, and 13% had myopia in accordance with referenced findings.

Our findings were consistent with the assertions of previous underreporting of cardiac involvement in MPS IVA according to the findings from the MorCAP population [10]. In our data, ECGs showed 47% had sinus arrhythmia and 35% had sinus tachycardia. Echocardiographic examinations also revealed 91% had valvular heart disease, as well as 73% and 50% had valvular regurgitation or stenosis, respectively. Nine patients (41%) had thick interventricular septum. The positive correlation between the presence of thick interventricular septum and increasing age reinforced the concept of the progressive nature of cardiac disease.

There are multifactorial etiologies for respiratory function impairment in patients with MPS IVA, including upper and lower airway obstruction, chest wall restriction, short stature, skeletal dysplasia, and cervical myelopathy. GAG is accumulated throughout the respiratory system [4]. In our study, we found a high prevalence of pulmonary function impairment by spirometric classification in MPS IVA patients characterized by 85% with small airway disease and 31% with restrictive lung disease. However, these percentages determined by comparing to normal standards for height which are problematic in MPS populations (severe short stature as well as joint disease makes height difficult to measure). Thus it would have expected that more than 31% to have restrictive lung disease.

Patients with MPS IVA experience hearing loss attributed to multiple causes. Conductive hearing loss is usually secondary to recurrent upper respiratory tract infection and serous otitis media, as well as caused by ossicle deformity. Sensorineural hearing loss may occur due to GAG accumulation. Most patients have a "mixed" hearing loss with the combination of conductive and sensorineural elements [10,32]. In our data, 67% patients had hearing loss, and a half of them could be classified with the mixed type, similar with those of the previous literature.

Patients with MPS have an increased risk of poor bone mineralization due to malnutrition, a particularly small frame, an abnormal gait, and reduction of physical activities caused by pain, poor health condition, or exercise intolerance [19]. In our study, according to BMD assessment by DXA, a half of MPS IVA patients had osteopenia or osteoporosis.

Sleep-disordered breathing, specifically obstructive sleep apnea, is a common disorder in patients with MPS [20]. In our study, only 3 MPS IVA patients had overnight polysomnographic reports, and all of them had some degree of obstructive sleep apnea.

Our study demonstrated that MPS IVA causes multi-systemic dysfunction, including skeletal, visual, auditory, cardiovascular, and respiratory systems, which severely affect patient quality of life by reducing endurance, increasing dependence on caregivers, and limiting participation in daily activities. As a result, multi-disciplinary approach to patient care is required once the diagnosis is made.

### Limitations

As a retrospective and multicenter study, there is a lack of complete data for all of our subjects. Assessments performed also varied by institution and treating physician. Small sample size reflected the rare nature of this genetic disorder, and the range of age was quite wide, as was the degree of disease severity. Therefore, studies in larger cohorts with a longer follow-up are warranted. However, our experience reflects the problem that clinicians are likely to encounter when treating patients with MPS IVA, since each patient presents with a significant variation.

## Conclusion

An understanding of the natural history and characterization of the clinical impairments involved in MPS IVA may allow early diagnosis and potentially facilitate better management of the disease. In our study, all affected Taiwanese patients experienced significant functional limitations, and regular evaluations and timely management may improve their quality of life. These findings and the follow-up data can be used to develop quality of care strategies, as well as to offer guidance for clinical trial outcomes and diagnostic process for patients with MPS IVA.

## Competing interests

Shuan-Pei Lin and Hsiang-Yu Lin have received honoraria for acting as consultants and study investigators for BioMarin Pharmaceutical Inc.

## Authors’ contributions

HYL and CKC performed acquisition, statistical analysis and interpretation of data, and drafting of the manuscript. SPL participated in design of the study, interpretation of the data and helped to draft the manuscript. CKC performed biochemical analyses and revised the manuscript. MRC, PCC, YYK, DMN, FJT, WLH, and JLL were responsible for patient screening. All authors read and accepted the manuscript.
